# 10-Methyl­isoalloxazine 5-oxide from synchrotron powder diffraction data

**DOI:** 10.1107/S1600536810048932

**Published:** 2010-11-27

**Authors:** Jan Rohlíček, Radek Cibulka, Jana Cibulková, Jaroslav Maixner, Michal Hušák

**Affiliations:** a Department of Solid State Chemistry, Institute of Chemical Technology in Prague, Technická 5, 166 28 Prague 6, Czech Republic; b Department of Organic Chemistry, Institute of Chemical Technology in Prague, Technická 5, 166 28 Prague 6, Czech Republic; c Central Laboratories, Institute of Chemical Technology in Prague, Technická 5, 166 28 Prague 6, Czech Republic

## Abstract

The title compound [systematic name: 10-methyl­benzo[*g*]pteridine-2,4(3*H*,10*H*)-dione 5-oxide], C_11_H_8_N_4_O_3_, consists of a large rigid isoalloxazine group which is approximately planar (r.m.s. deviation = 0.037 Å). In the crystal, inter­molecular N—H⋯O hydrogen bonds link the mol­ecules into centrosymmetric dimers. Dimers related by translation along the *c* axis form stacks through π–π inter­actions [centroid–centroid distances = 3.560 (5) and 3.542 (5) Å]. Weak inter­molecular C—H⋯O inter­actions further consolidate the crystal packing.

## Related literature

For the preparation of the title compound, see: Yoneda *et al.* (1976 ▶). For background to flavins, see: Massey (2000 ▶), Palfey & Massey (1998 ▶); Müller (1991 ▶). For a description of the Cambridge Structural Database, see: Allen (2002 ▶). For the crystal structures of similar compounds, see: Wang & Fritchie (1973 ▶); Farrán *et al.* (2007 ▶). 
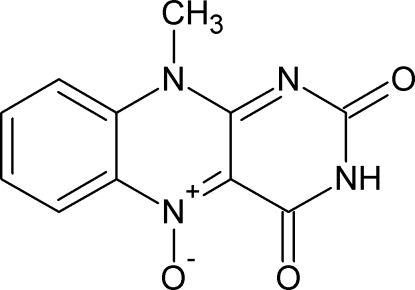


## Experimental

### 

#### Crystal data

C_11_H_8_N_4_O_3_
*M* *_r_* = 244.21
Monoclinic, 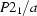

*a* = 13.8774 (6) Å
*b* = 14.5321 (4) Å
*c* = 4.9305 (2) Å
β = 90.830 (3)°
*V* = 994.22 (5) Å^3^
*Z* = 4
Synchrotron radiation
λ = 0.8856 Å
μ = 0.20 mm^−1^
*T* = 293 K
cylinder, 20 × 1 mm

#### Data collection

ESRF Grenoble, BM20 diffractometer
Specimen mounting: capilary
Data collection mode: transmission
Scan method: step
2θ_min_ = 4.0°, 2θ_max_ = 36.5°, 2θ_step_ = 0.01°

#### Refinement

*R*_p_ = 0.042
*R*_wp_ = 0.056
*R*_exp_ = 0.021
*R*_Bragg_ = 0.06
*R*(*F* ^2^) = 0.060
χ^2^ = 7.129
3251 data points
73 parameters
57 restraints
H-atom parameters not refined

### 

Data collection: *ESRF SPEC* (Certified Scientific Software, 2003 ▶); cell refinement: *GSAS* (Larson & Von Dreele, 1994 ▶); data reduction: *CRYSFIRE* (Shirley, 2000 ▶); program(s) used to solve structure: *FOX* (Favre-Nicolin & Černý, 2002 ▶); program(s) used to refine structure: *GSAS*; molecular graphics: *Mercury* (Macrae *et al.*, 2006 ▶) and *PLATON* (Spek, 2009 ▶); software used to prepare material for publication: *publCIF* (Westrip, 2010 ▶).

## Supplementary Material

Crystal structure: contains datablocks global, I. DOI: 10.1107/S1600536810048932/cv5002sup1.cif

Rietveld powder data: contains datablocks I. DOI: 10.1107/S1600536810048932/cv5002Isup2.rtv

Additional supplementary materials:  crystallographic information; 3D view; checkCIF report

## Figures and Tables

**Table 1:** Hydrogen-bond geometry (Å, °)

*D*—H⋯*A*	*D*—H	H⋯*A*	*D*⋯*A*	*D*—H⋯*A*
N3—H1*N*3⋯O11^i^	0.86	1.92	2.764 (14)	166
C14—H2*C*14⋯O12^ii^	0.95	2.63	3.097 (16)	111
C14—H1*C*14⋯O13^iii^	0.95	2.33	3.194 (17)	151

Symmetry codes: (i) 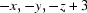; (ii) 
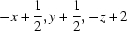
; (iii) 
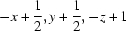
.

**Fig. 1.**
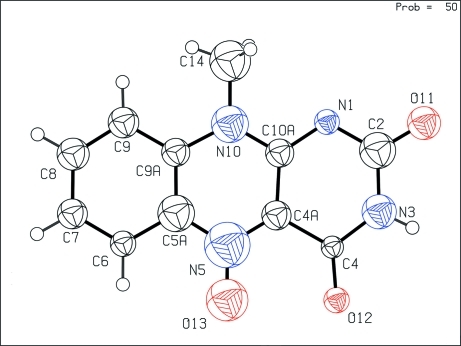
ORTEP plot of the title molecule with the displacement ellipsoids drawn at the 50% probability level. The H atoms are shown as spheres of arbitrary radius.

**Fig. 2.**
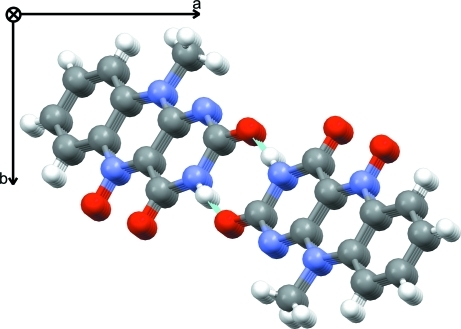
View of N—H···O hydrogen bonded (dotted lines) centrosymmetric dimers in the title crystal structure.

**Fig. 3.**
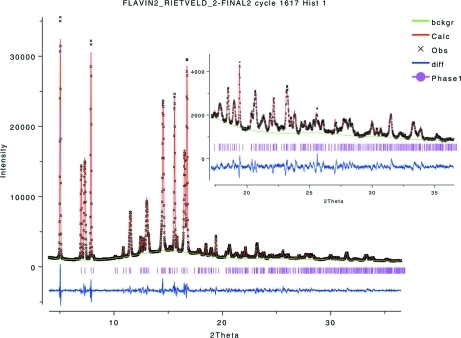
The final Rietveld plot showing the measured data (black thin-cross), calculated data (red line) and difference curve (blue line). Calculated positions of the reflection are shown by vertical bars.

## supplementary crystallographic information

### Comment

The titled compound, 10-methylbenzo[*g*]pteridine-2,4(3*H*,10H)-dione-5-oxide belongs to a group of isoalloxazine-5-oxides which are important intermediates in synthesis of flavin derivatives (Yoneda *et al.*, 1976). Flavins are important natural compounds which act as cofactors in redox enzymes (Massey, 2000; Palfey & Massey, 1998; Müller, 1991). Synthetic procedure utilizing isoalloxazine-5-oxides allows synthesis of non-natural flavin derivatives which are used in flavoenzyme models. To our knowledge, no crystal structure of any isoalloxazine-5-oxide has so far been published.

The asymmetric unit contains one molecule of the title compound, which is almost planar. The molecule consists of a isoalloxazine group which is formed by three connected rings - benzene, pyrazine and uracil ring. The most significant deviation from planarity occurs at the uracil ring, where the oxygen atom O12 is found to be out of the plane (torsion angle C10*a*—C4a—C4—O12 is app. 6.5°). The deviation of O12 atom is in accordance with the C4 carbon atom position which is slightly out of the plane and form the planar *sp*^2^ hybridization. The next deviation from planarity is on the pyrazine ring where the nitrogen atom N10 leaves out of the plane and the connected methyl group follow the direction of *sp*^2^ hybridization (torsion angle C4a—C10*a*—N10—C14 is app. 5.5°). Molecules of titled compound are connected together by several hydrogen bonds (Table 2) and by π-π interactions (Table 1). The strongest hydrogen bond N3-H1N3···O11 connects always two molecules together into dimers, see Fig. 2. On the other hand, the other two hydrogen bonds C14-H1C14···O13 and C14-H2C14···O12 together with π-π interactions form molecules to the infinity layers which are parallel to (100). These layers are connected by the above mentioned N3-H1N3···O11 hydrogen bonds.

The survey in the CSD (Allen, 2002) found several crystal structures of similar molecules which are derived from isoalloxazine, but no crystal structure of isoalloxazine-5-oxide which we present here was found. The similar crystal structures of 10-Methylisoalloxazine (Wang & Fritchie, 1973) and 7,10-Dimethylisoalloxazine (Farrán *et al.*, 2007) can be used for comparison. In both structures the isoalloxazine part is approximately planar and both structures form dimers which are connected by N—H···O hydrogen bonds. The occurrence of the π-π stacking is also evident.

### Experimental

The title compound was prepared according to Yoneda *et al.* (1976). The 6-(*N*-Methylanilino)uracil (15.6 g; 65 mmol) was dissolved in acetic acid (130 ml) and sodium nitrite (22.8 g, 0.325 mol) was added. The mixture was stirred at room temperature for 3 h, diluted with water (325 ml), and allowed to stand overnight. The crystals were collected by filtration, washed with water several times, and dried. Recrystallization from aqueous acetic acid gave orange needles (17.4 g; 98%). *M*.p. >300 °C.

^1^H NMR (DMSO-d_6_, 300 MHz) δ3,89 (s, 3H, –C*H*_3_), 7,57 (m, 1H, Ar—H), 7.95 (m, 2H, Ar—H), 8,30 (d, 1H, J=8.2, Ar—H), 11.11 (s, 1H, N*H*).

For C_11_H_8_N_4_O_3_ (244.21) calculated: 54.10% C, 3.30% H, 22.94% N; found: 54.18% C, 3.41% H, 23.05% N.

The X-Ray diffraction data were collected on the Rossendorf Beamline BM20 at the ESRF in Grenoble. The energy was fixed at 14 keV which is equal to λ=0.8856 Å wavelength (the precise wavelength value was confirmed by the LaB_6_ standard measurement). The beamline was equipped with double-crystal Si(111) monochromator and with two collimating/focusing mirrors (Si and Pt-coating). The sample was placed in the 1-mm-borosilicate glass capillary rotated during the measurement. The diffraction pattern was measured at room temperature from 4° 2θ to 36.5° 2θ with the 0.01° 2θ step size.

### Refinement

The indexation was performed by the CRYSFIRE package (Shirley, 2000). The final cell *a*=13.8774 (6) Å, *b* = 14.5321 (4) Å, *c* = 4.9305 (2) Å, *β* = 90.830 (3) ° and V = 993.48 (7) Å^3^ was found from 20 peaks by several embedded indexation programs. If the volume of the molecule is compared with the volume of the found unit, it is clear that there are four molecules in the unit cell. The space group *P*2_1_/*a* (*Z* = 4) was selected according to the peak extinction and the agreement of the Le-bail fit. The crystal structure was solved by parallel tempering algorithm implemented in the program FOX (Favre-Nicolin & Černý, 2002). We decided to test the structure solution run for other space groups which had similar peak extinction to validate the selection of the P21/a space group. These two space groups P 2/m and P21/m were selected, but the solution was not found.

The final refinement was performed with *GSAS* (Larson & Von Dreele, 1994). The structure was restrained by soft bonds and angles restraints. Four planar groups restraints were added - one for the benzene ring (C5a—C9a) and remaining three for the *sp*^2^ hybridization (C2/N1/N3/O11, C4a/C4/N3/O12 and C10*a*/C4a/C4/N5). At the final stage, positions and isotropic thermal parameters of all non-hydrogen atoms were refined to the low agreement *R*-factors (*R_p_* = 4.2%, *R_wp_* = 5.6%). During the refinement all hydrogen atoms were kept in their theoretical positions and were not refined. The final Rietveld plot is shown on the Fig. 3.

### Crystal data

**Table:**

C_11_H_8_N_4_O_3_	*Z* = 4
*M**_r_* = 244.21	*F*(000) = 504
Monoclinic, *P*2_1_/*a*	*D*_x_ = 1.633 Mg m^−^^3^
Hall symbol: -P 2yab	Synchrotron radiation, λ = 0.8856 Å
*a* = 13.8774 (6) Å	µ = 0.20 mm^−^^1^
*b* = 14.5321 (4) Å	*T* = 293 K
*c* = 4.9305 (2) Å	yellow
β = 90.830 (3)°	cylinder, 20 × 1 mm
*V* = 994.22 (5) Å^3^	

### Data collection

**Table:**

ESRF Grenoble, BM20 diffractometer	Data collection mode: transmission
Radiation source: synchrotron	Scan method: step
Specimen mounting: capilary	2θ_min_ = 4.0°, 2θ_max_ = 36.5°, 2θ_step_ = 0.01°

### Refinement

**Table:**

Least-squares matrix: full	73 parameters
*R*_p_ = 0.042	57 restraints
*R*_wp_ = 0.056	0 constraints
*R*_exp_ = 0.021	Hydrogen site location: inferred from neighbouring sites
*R*_Bragg_ = 0.06	H-atom parameters not refined
*R*(*F*^2^) = 0.06000	Weighting scheme based on measured s.u.'s
χ^2^ = 7.129	(Δ/σ)_max_ = 0.02
3251 data points	Background function: GSAS Background function number 1 with 20 terms. Shifted Chebyshev function of 1st kind 1: 1199.79 2: -234.522 3: -315.536 4: 152.956 5: 123.532 6: -246.657 7: 116.810 8: 83.9272 9: -107.809 10: -12.4938 11: 79.2500 12: -25.2804 13: -27.8174 14: 13.6120 15: 6.03858 16: -3.86487 17: 2.09281 18: 9.92947 19: -18.6000 20: 1.36657
Excluded region(s): none	Preferred orientation correction: March-Dollase AXIS 1 Ratio= 0.89956 h= 0.000 k= 0.000 l= 1.000 Prefered orientation correction range: Min= 0.85318, Max= 1.37377
Profile function: CW Profile function number 4 with 21 terms Pseudovoigt profile coefficients as parameterized in P. Thompson, D.E. Cox & J.B. Hastings (1987). J. Appl. Cryst.,20,79-83. Asymmetry correction of L.W. Finger, D.E. Cox & A. P. Jephcoat (1994). J. Appl. Cryst.,27,892-900. Microstrain broadening by P.W. Stephens, (1999). J. Appl. Cryst.,32,281-289. #1(GU) = 118.875 #2(GV) = 80.014 #3(GW) = 0.010 #4(GP) = 0.000 #5(LX) = 1.385 #6(ptec) = 0.00 #7(trns) = 0.00 #8(shft) = 0.0000 #9(sfec) = 0.00 #10(S/L) = 0.0005 #11(H/L) = 0.0142 #12(eta) = 0.0000 #13(S400 ) = 1.7E-01 #14(S040 ) = 1.8E-02 #15(S004 ) = 6.2E-01 #16(S220 ) = -4.6E-02 #17(S202 ) = 3.4E-01 #18(S022 ) = 1.6E-01 #19(S301 ) = -5.6E-01 #20(S103 ) = 7.9E-01 #21(S121 ) = 7.0E-02 Peak tails are ignored where the intensity is below 0.0001 times the peak Aniso. broadening axis 0.0 0.0 1.0	

### Fractional atomic coordinates and isotropic or equivalent isotropic displacement parameters (Å^2^)

**Table:**

	*x*	*y*	*z*	*U*_iso_*/*U*_eq_	
N1	0.0773 (7)	0.1443 (5)	1.0053 (18)	0.036 (6)*	
C2	0.0483 (8)	0.0827 (7)	1.194 (3)	0.075 (8)*	
N3	0.0977 (7)	−0.0028 (6)	1.2303 (19)	0.068 (6)*	
C4	0.1784 (5)	−0.0297 (5)	1.0946 (14)	0.028 (8)*	
C4a	0.2075 (5)	0.0367 (5)	0.8768 (14)	0.049 (7)*	
N5	0.2843 (7)	0.0175 (6)	0.725 (2)	0.120 (8)*	
C5a	0.3110 (6)	0.0833 (6)	0.5319 (18)	0.077 (9)*	
C6	0.3917 (7)	0.0645 (6)	0.365 (2)	0.037 (7)*	
C7	0.4210 (6)	0.1290 (8)	0.180 (2)	0.052 (7)*	
C8	0.3703 (8)	0.2112 (7)	0.1520 (18)	0.059 (8)*	
C9	0.2917 (7)	0.2332 (6)	0.311 (2)	0.054 (8)*	
C9a	0.2610 (6)	0.1684 (6)	0.5019 (18)	0.045 (9)*	
N10	0.1787 (7)	0.1824 (7)	0.6664 (19)	0.078 (7)*	
C10a	0.1522 (5)	0.1216 (5)	0.8572 (14)	0.046 (8)*	
O11	−0.0238 (7)	0.1000 (7)	1.338 (2)	0.063 (5)*	
O12	0.2230 (7)	−0.1014 (5)	1.1524 (17)	0.037 (4)*	
O13	0.3291 (8)	−0.0597 (7)	0.748 (2)	0.100 (5)*	
C14	0.1211 (10)	0.2660 (9)	0.619 (3)	0.095 (9)*	
H1N3	0.0746	−0.0405	1.3466	0.0804*	
H1C6	0.425	0.0076	0.3816	0.0456*	
H1C7	0.4758	0.1173	0.0718	0.0612*	
H1C8	0.3906	0.254	0.0188	0.0708*	
H1C9	0.2597	0.2905	0.2901	0.0636*	
H1C14	0.1488	0.3013	0.479	0.114*	
H2C14	0.119	0.3013	0.781	0.114*	
H3C14	0.057	0.249	0.567	0.114*	

### Geometric parameters (Å, °)

**Table:**

O11—C2	1.261 (16)	N10—C9a	1.425 (13)
C4—C4a	1.503 (10)	N3—C2	1.429 (14)
O12—C4	1.243 (11)	N3—C4	1.370 (12)
C5a—C9a	1.425 (12)	N5—C5a	1.403 (13)
O13—N5	1.287 (14)	N5—C4a	1.341 (12)
C5a—C6	1.426 (13)	C10a—C4a	1.455 (10)
N1—C10a	1.321 (12)	C14—H1C14	0.9500
C6—C7	1.374 (14)	C14—H2C14	0.9500
N1—C2	1.356 (15)	C14—H3C14	0.9500
C7—C8	1.392 (15)	C6—H1C6	0.9500
N10—C10a	1.346 (12)	C7—H1C7	0.9500
C8—C9	1.390 (14)	N3—H1N3	0.8600
N10—C14	1.471 (17)	C8—H1C8	0.9500
C9—C9a	1.402 (13)	C9—H1C9	0.9500
			
Cg1···Cg2^i^	3.56 (1)	Cg1···Cg3^i^	3.54 (1)
			
C2—N1—C10A	117.3 (8)	C8—C9—C9A	118.2 (8)
C2—N3—C4	125.5 (9)	N10—C9A—C5A	117.3 (8)
O13—N5—C4A	121.3 (9)	N10—C9A—C9	122.7 (8)
O13—N5—C5A	121.5 (9)	C5A—C9A—C9	120.0 (8)
C4A—N5—C5A	117.2 (8)	N1—C10A—N10	116.6 (8)
C9A—N10—C10A	122.3 (9)	N1—C10A—C4A	126.4 (7)
C9A—N10—C14	117.8 (9)	N10—C10A—C4A	117.0 (7)
C10A—N10—C14	120.0 (9)	C2—N3—H1N3	117
O11—C2—N1	120.0 (10)	C4—N3—H1N3	117
O11—C2—N3	119.1 (11)	C5A—C6—H1C6	120
N1—C2—N3	120.9 (10)	C7—C6—H1C6	120
O12—C4—N3	122.4 (8)	C6—C7—H1C7	120
O12—C4—C4A	124.3 (7)	C8—C7—H1C7	120
N3—C4—C4A	113.3 (7)	C7—C8—H1C8	118
N5—C4A—C4	119.3 (7)	C9—C8—H1C8	119
N5—C4A—C10A	124.2 (7)	C8—C9—H1C9	121
C4—C4A—C10A	116.4 (6)	C9A—C9—H1C9	121
N5—C5A—C6	118.6 (8)	N10—C14—H1C14	110
N5—C5A—C9A	121.9 (8)	N10—C14—H2C14	110
C6—C5A—C9A	119.5 (8)	N10—C14—H3C14	109
C5A—C6—C7	119.7 (8)	H1C14—C14—H2C14	110
C6—C7—C8	119.8 (9)	H1C14—C14—H3C14	109
C7—C8—C9	122.8 (9)		

Symmetry codes:  (i) *x*, *y*, *z*+1.

### Hydrogen-bond geometry (Å, °)

**Table:**

*D*—H···*A*	*D*—H	H···*A*	*D*···*A*	*D*—H···*A*
N3—H1N3···O11^ii^	0.86	1.92	2.764 (14)	166
C14—H2C14···O12^iii^	0.95	2.63	3.097 (16)	111
C14—H1C14···O13^iv^	0.95	2.33	3.194 (17)	151

Symmetry codes: (ii) −*x*, −*y*, −*z*+3; (iii) −*x*+1/2, *y*+1/2, −*z*+2; (iv) −*x*+1/2, *y*+1/2, −*z*+1.
